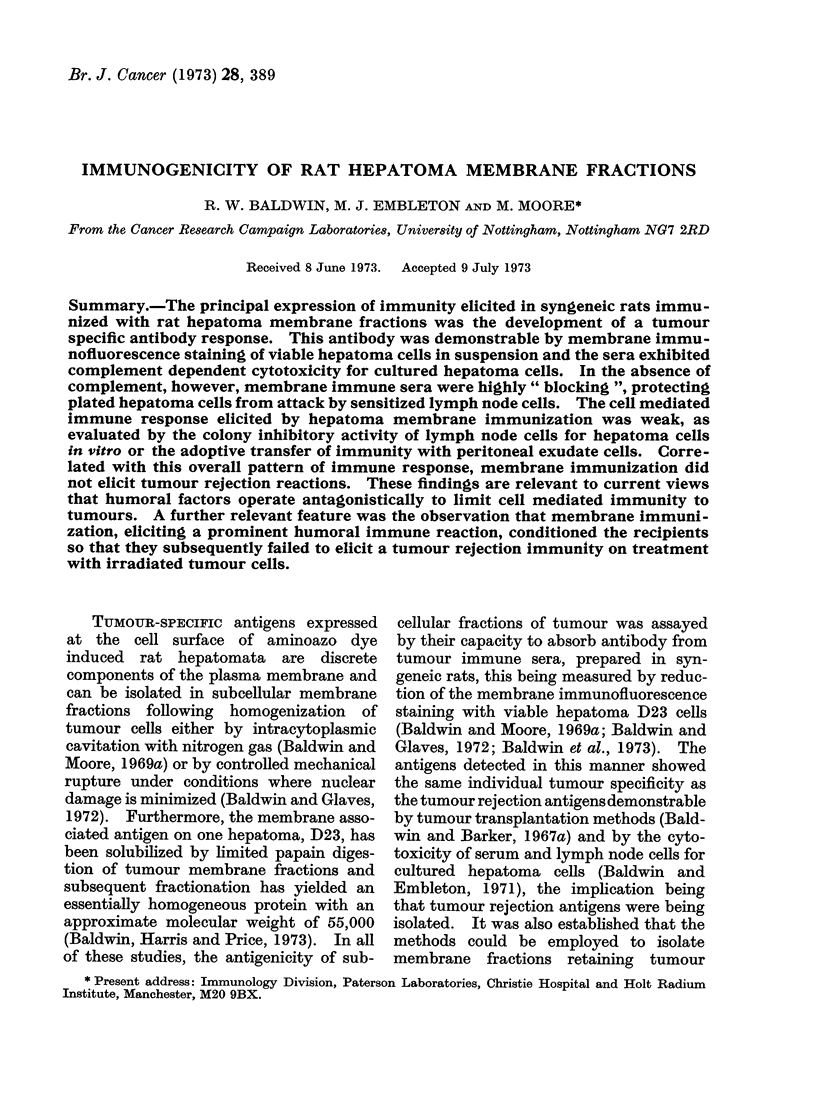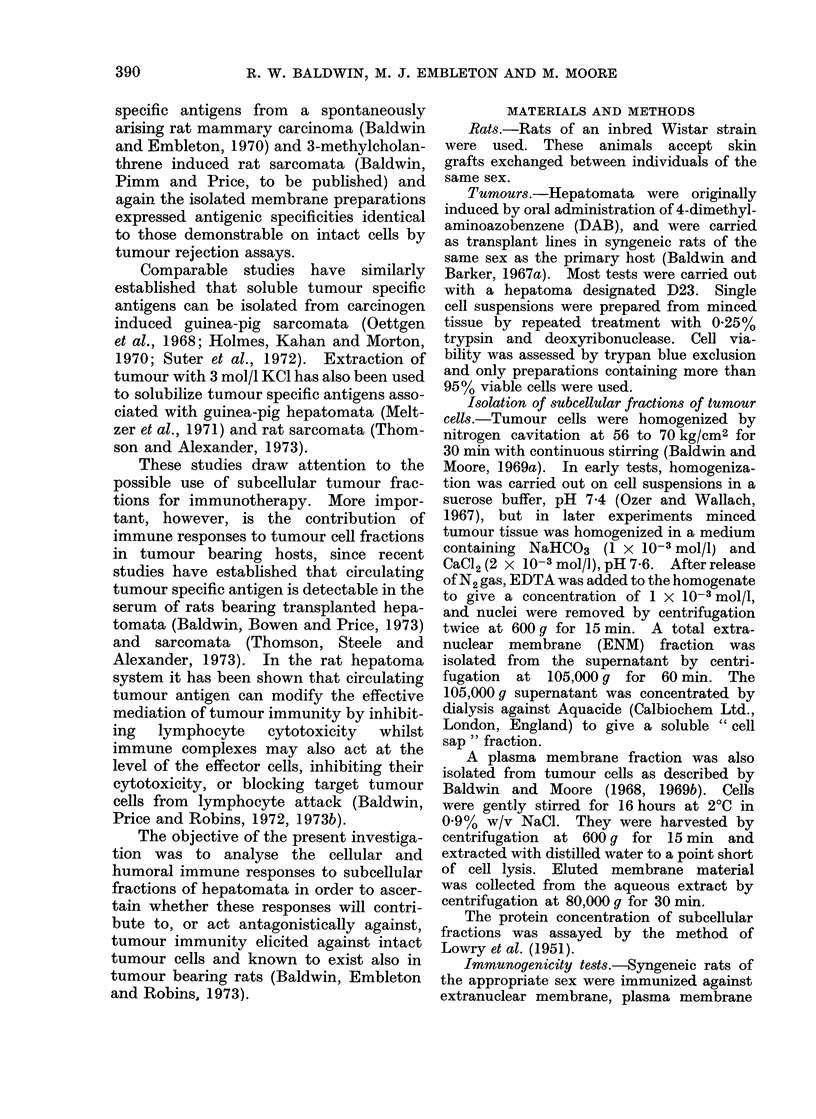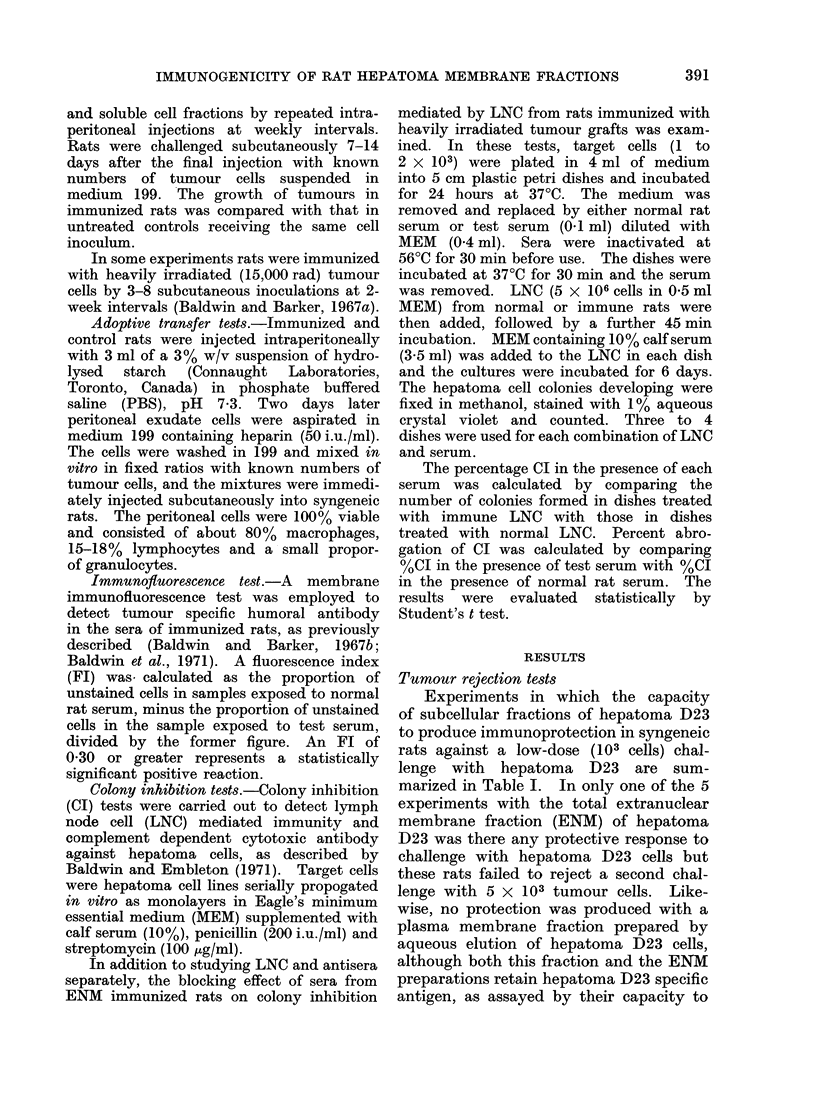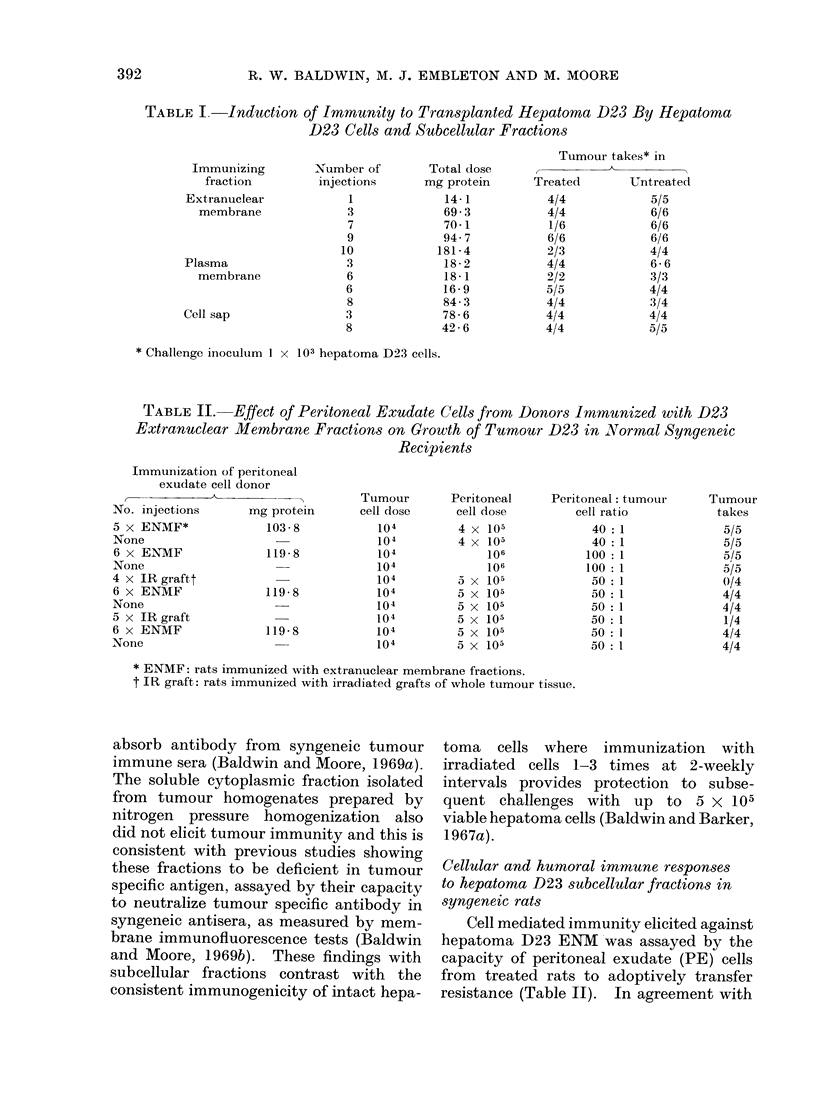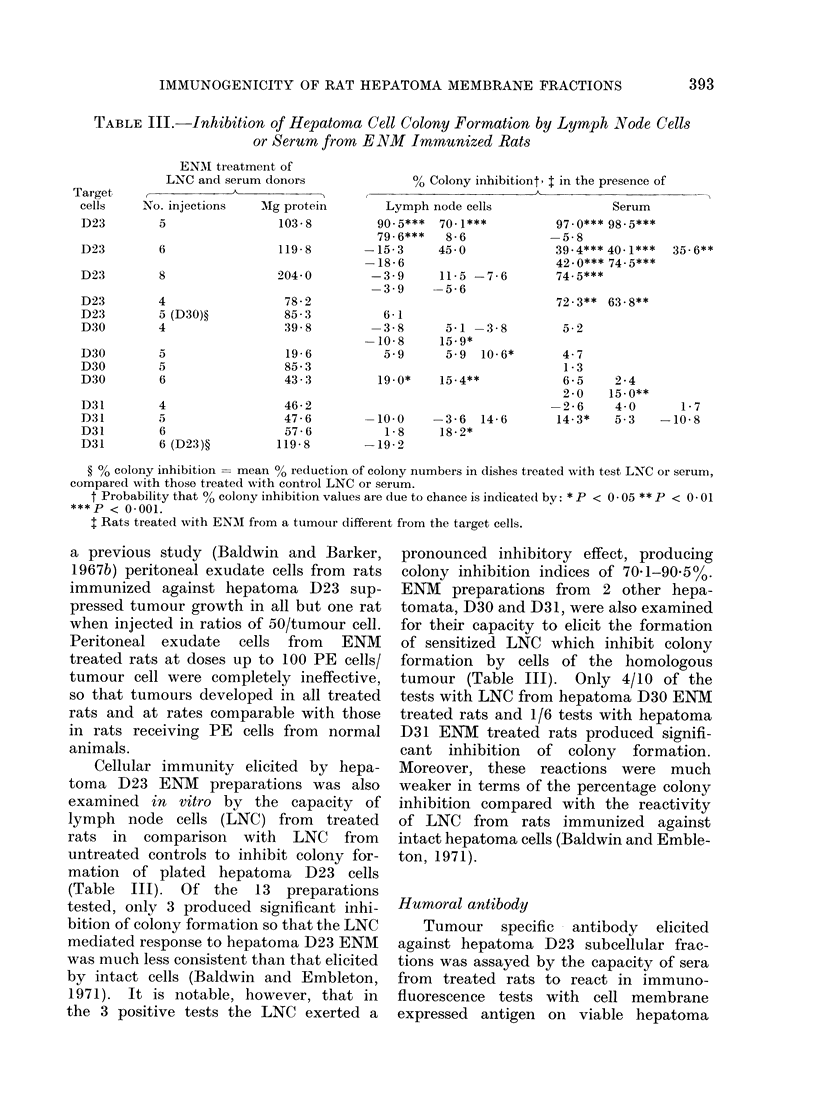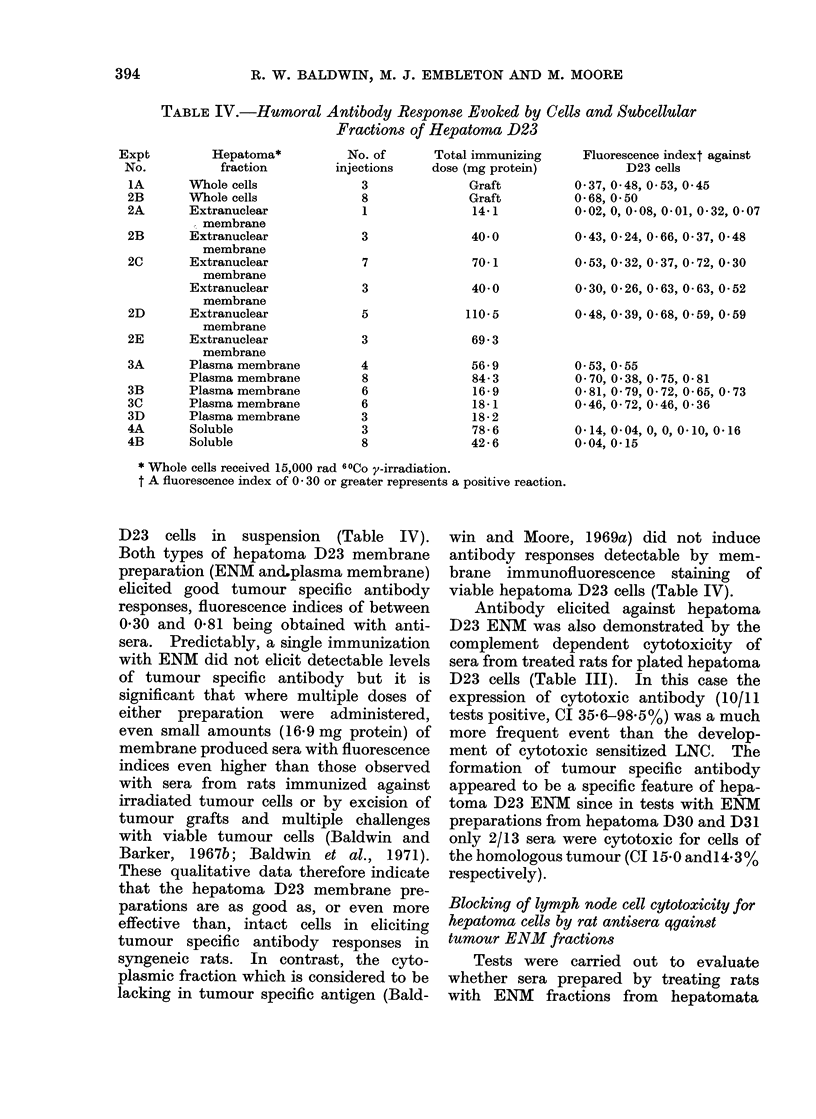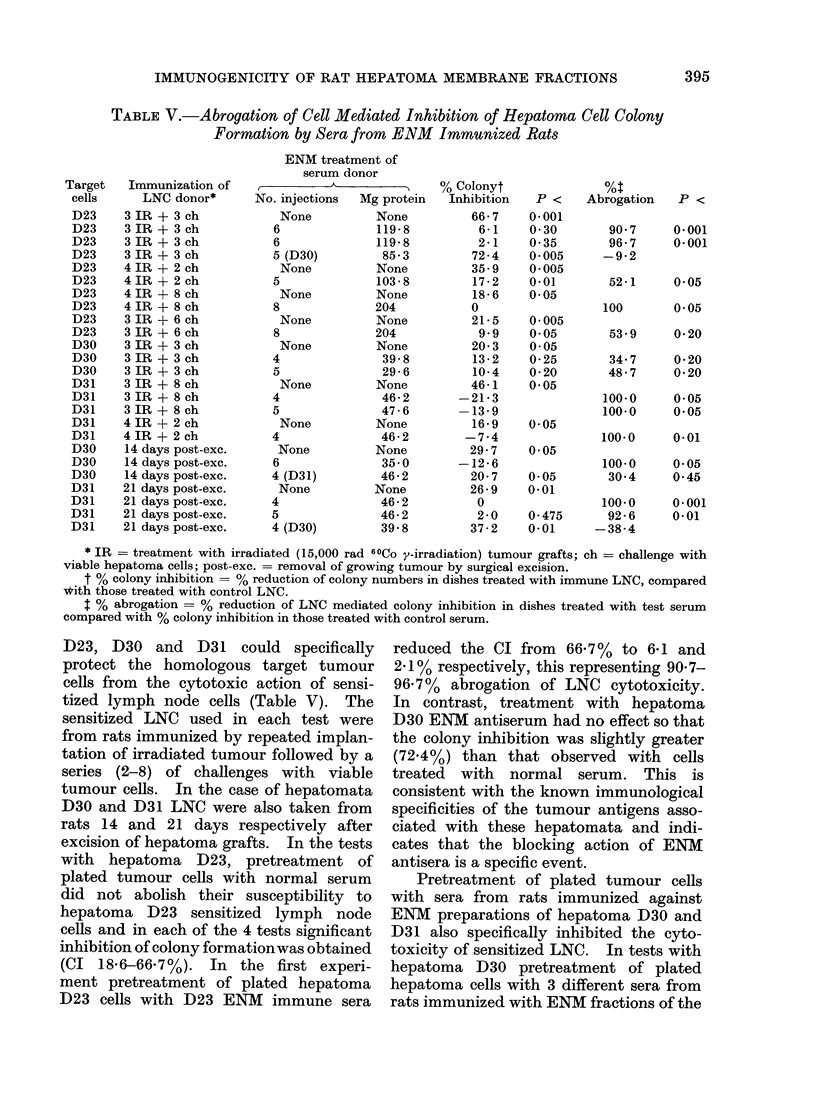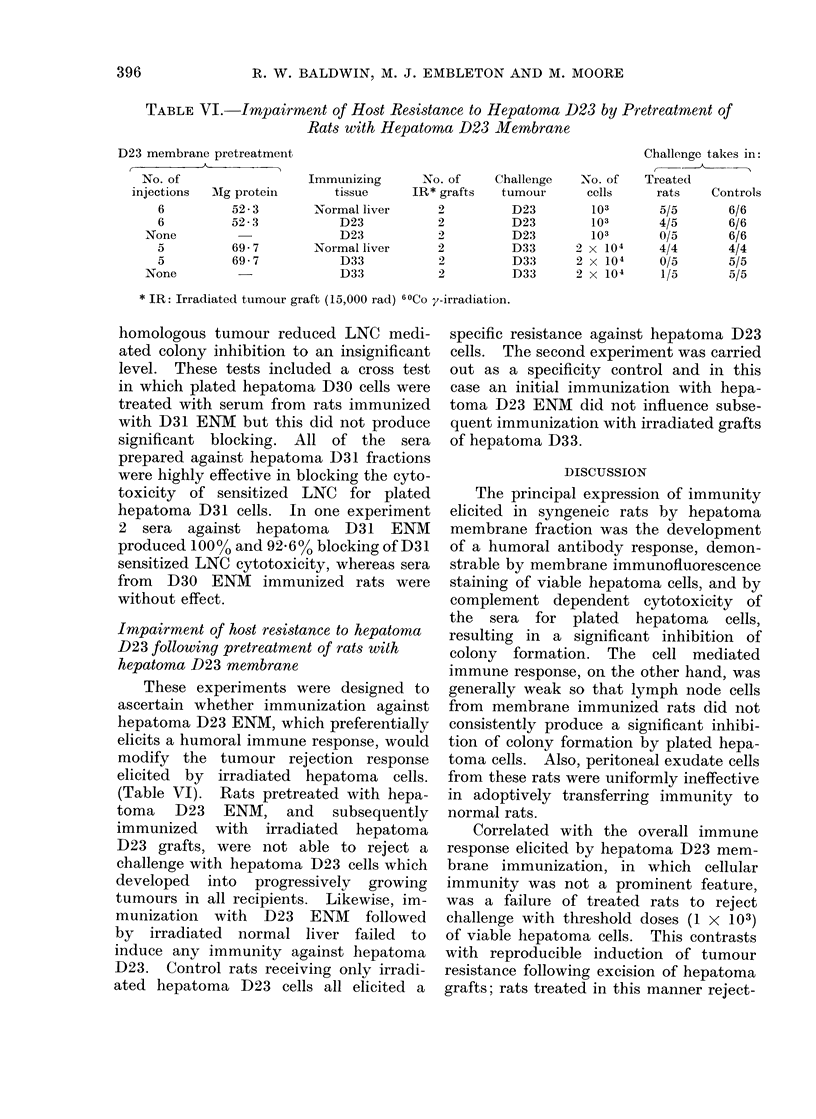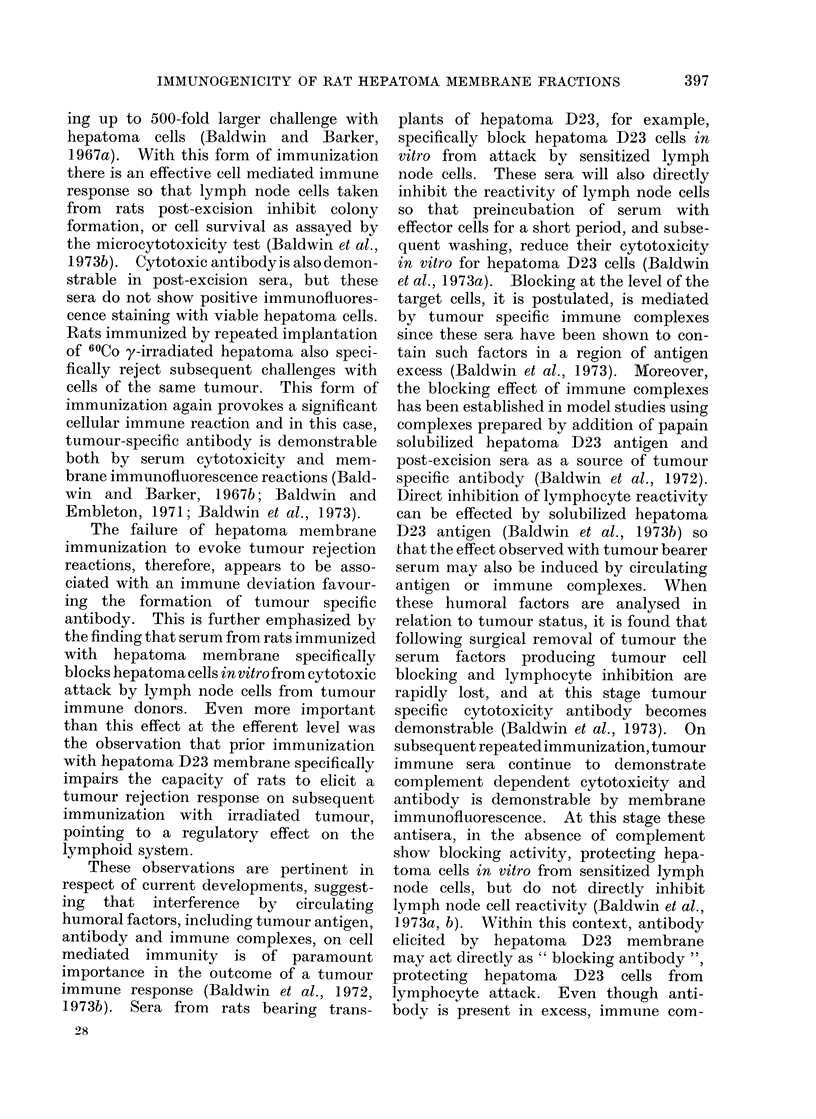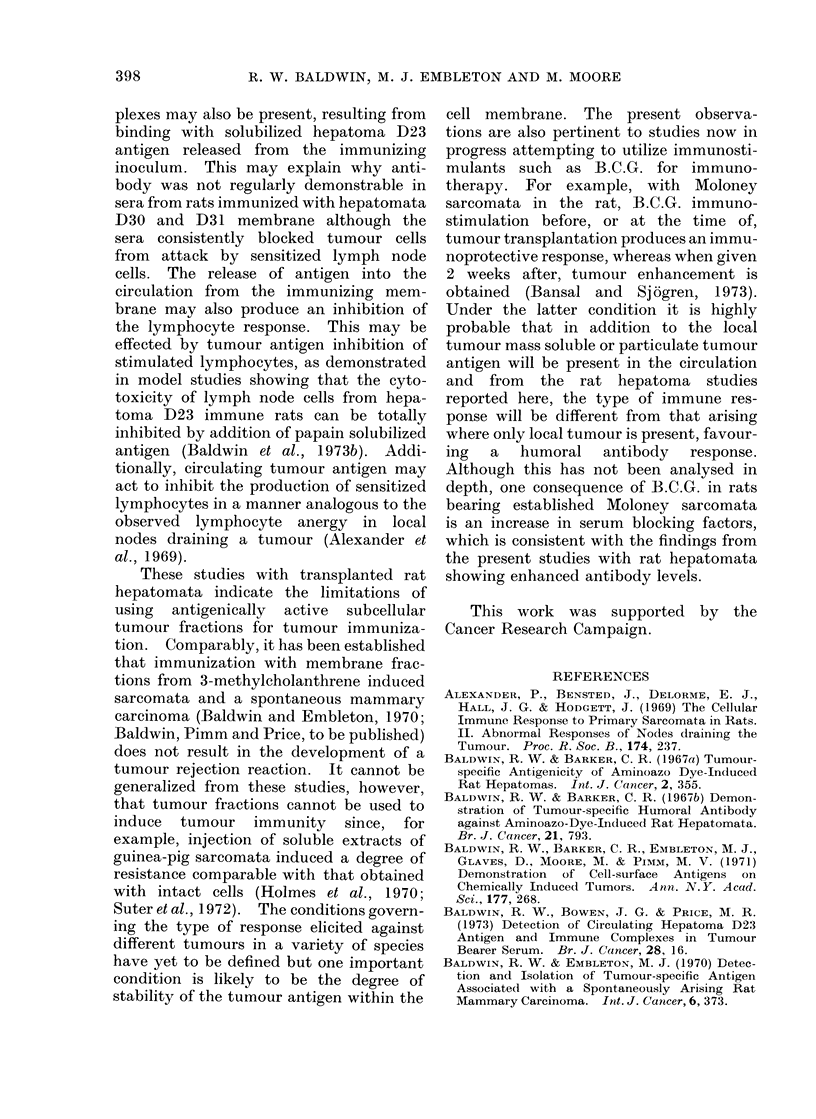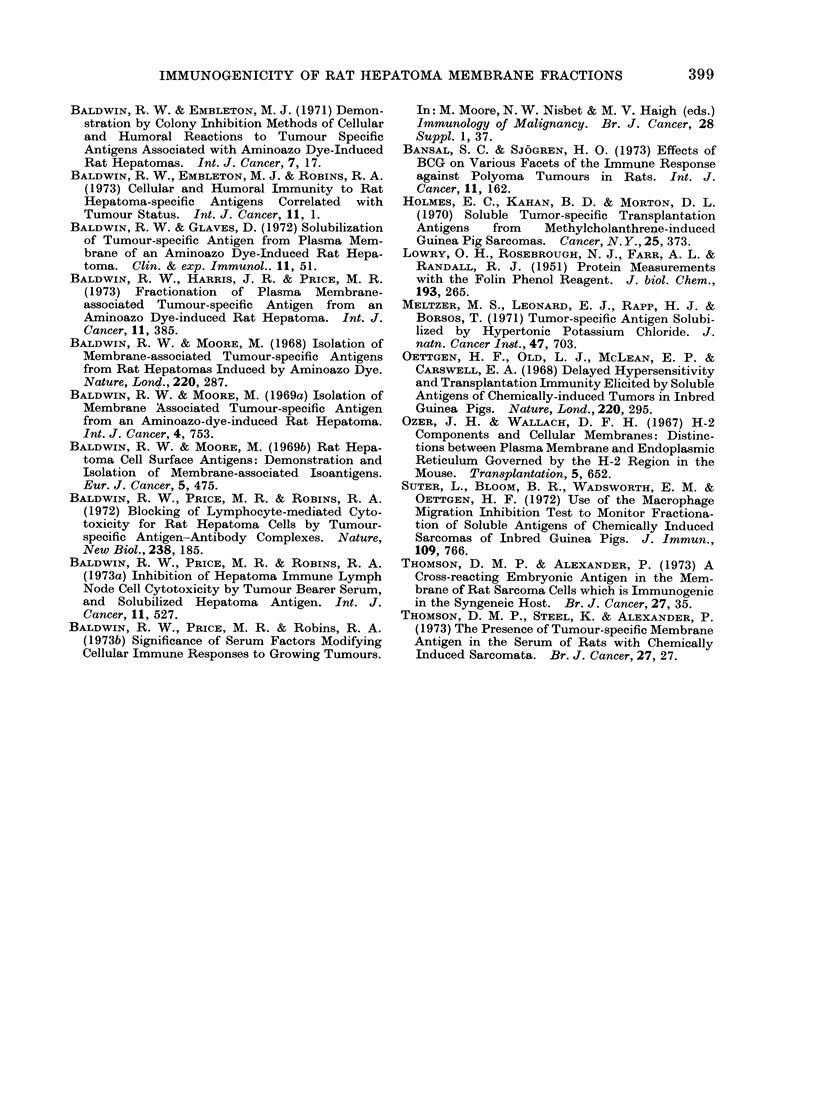# Immunogenicity of Rat Hepatoma Membrane Fractions

**DOI:** 10.1038/bjc.1973.164

**Published:** 1973-11

**Authors:** R. W. Baldwin, M. J. Embleton, M. Moore

## Abstract

The principal expression of immunity elicited in syngeneic rats immunized with rat hepatoma membrane fractions was the development of a tumour specific antibody response. This antibody was demonstrable by membrane immunofluorescence staining of viable hepatoma cells in suspension and the sera exhibited complement dependent cytotoxicity for cultured hepatoma cells. In the absence of complement, however, membrane immune sera were highly “blocking”, protecting plated hepatoma cells from attack by sensitized lymph node cells. The cell mediated immune response elicited by hepatoma membrane immunization was weak, as evaluated by the colony inhibitory activity of lymph node cells for hepatoma cells *in vitro* or the adoptive transfer of immunity with peritoneal exudate cells. Correlated with this overall pattern of immune response, membrane immunization did not elicit tumour rejection reactions. These findings are relevant to current views that humoral factors operate antagonistically to limit cell mediated immunity to tumours. A further relevant feature was the observation that membrane immunization, eliciting a prominent humoral immune reaction, conditioned the recipients so that they subsequently failed to elicit a tumour rejection immunity on treatment with irradiated tumour cells.


Br. J. Cancer (1973) 28, 389

### IMMUNOGENICITY OF RAT HEPATOMA MEMBRANE FRACTIONS

R. W. BALDWIN, M. J. EMBLETON AND M. MOORE*

*From the Cancer Research Campaign Laboratories, University of Nottingham, Nottingham NG7 2RD*

Received 8 June 1973. Accepted 9 July 1973

**Summary.—The principal expression of immunity elicited in syngeneic rats immunized with rat hepatoma membrane fractions was the development of a tumour specific antibody response. This antibody was demonstrable by membrane immunofluorescence staining of viable hepatoma cells in suspension and the sera exhibited complement dependent cytotoxicity for cultured hepatoma cells. In the absence of complement, however, membrane immune sera were highly " blocking ", protecting plated hepatoma cells from attack by sensitized lymph node cells. The cell mediated immune response elicited by hepatoma membrane immunization was weak, as evaluated by the colony inhibitory activity of lymph node cells for hepatoma cells *in vitro* or the adoptive transfer of immunity with peritoneal exudate cells. Correlated with this overall pattern of immune response, membrane immunization did not elicit tumour rejection reactions. These findings are relevant to current views that humoral factors operate antagonistically to limit cell mediated immunity to tumours. A further relevant feature was the observation that membrane immunization, eliciting a prominent humoral immune reaction, conditioned the recipients so that they subsequently failed to elicit a tumour rejection immunity on treatment with irradiated tumour cells.**

TUMOUR-SPECIFIC antigens expressed at the cell surface of aminoazo dye induced rat hepatomata are discrete components of the plasma membrane and can be isolated in subcellular membrane fractions following homogenization of tumour cells either by intracytoplasmic cavitation with nitrogen gas (Baldwin and Moore, 1969*a*) or by controlled mechanical rupture under conditions where nuclear damage is minimized (Baldwin and Glaves, 1972). Furthermore, the membrane associated antigen on one hepatoma, D23, has been solubilized by limited papain digestion of tumour membrane fractions and subsequent fractionation has yielded an essentially homogeneous protein with an approximate molecular weight of 55,000 (Baldwin, Harris and Price, 1973). In all of these studies, the antigenicity of subcellular fractions of tumour was assayed by their capacity to absorb antibody from tumour immune sera, prepared in syngeneic rats, this being measured by reduction of the membrane immunofluorescence staining with viable hepatoma D23 cells (Baldwin and Moore, 1969*a*; Baldwin and Glaves, 1972; Baldwin *et al.*, 1973). The antigens detected in this manner showed the same individual tumour specificity as the tumour rejection antigens demonstrable by tumour transplantation methods (Baldwin and Barker, 1967*a*) and by the cytotoxicity of serum and lymph node cells for cultured hepatoma cells (Baldwin and Embleton, 1971), the implication being that tumour rejection antigens were being isolated. It was also established that the methods could be employed to isolate membrane fractions retaining tumour

* Present address: Immunology Division, Paterson Laboratories, Christie Hospital and Holt Radium Institute, Manchester, M20 9BX.

specific antigens from a spontaneously arising rat mammary carcinoma (Baldwin and Embleton, 1970) and 3-methylcholanthrene induced rat sarcomata (Baldwin, Pimm and Price, to be published) and again the isolated membrane preparations expressed antigenic specificities identical to those demonstrable on intact cells by tumour rejection assays.

Comparable studies have similarly established that soluble tumour specific antigens can be isolated from carcinogen induced guinea-pig sarcomata (Oettgen *et al.*, 1968; Holmes, Kahan and Morton, 1970; Suter *et al.*, 1972). Extraction of tumour with 3 mol/l KCl has also been used to solubilize tumour specific antigens associated with guinea-pig hepatomata (Meltzer *et al.*, 1971) and rat sarcomata (Thomson and Alexander, 1973).

These studies draw attention to the possible use of subcellular tumour fractions for immunotherapy. More important, however, is the contribution of immune responses to tumour cell fractions in tumour bearing hosts, since recent studies have established that circulating tumour specific antigen is detectable in the serum of rats bearing transplanted hepatomata (Baldwin, Bowen and Price, 1973) and sarcomata (Thomson, Steele and Alexander, 1973). In the rat hepatoma system it has been shown that circulating tumour antigen can modify the effective mediation of tumour immunity by inhibiting lymphocyte cytotoxicity whilst immune complexes may also act at the level of the effector cells, inhibiting their cytotoxicity, or blocking target tumour cells from lymphocyte attack (Baldwin, Price and Robins, 1972, 1973*b*).

The objective of the present investigation was to analyse the cellular and humoral immune responses to subcellular fractions of hepatomata in order to ascertain whether these responses will contribute to, or act antagonistically against, tumour immunity elicited against intact tumour cells and known to exist also in tumour bearing rats (Baldwin, Embleton and Robins, 1973).

### MATERIALS AND METHODS

*Rats.*—Rats of an inbred Wistar strain were used. These animals accept skin grafts exchanged between individuals of the same sex.

*Tumours.*—Hepatomata were originally induced by oral administration of 4-dimethylaminoazobenzene (DAB), and were carried as transplant lines in syngeneic rats of the same sex as the primary host (Baldwin and Barker, 1967*a*). Most tests were carried out with a hepatoma designated D23. Single cell suspensions were prepared from minced tissue by repeated treatment with 0·25% trypsin and deoxyribonuclease. Cell viability was assessed by trypan blue exclusion and only preparations containing more than 95% viable cells were used.

*Isolation of subcellular fractions of tumour cells.*—Tumour cells were homogenized by nitrogen cavitation at 56 to 70 kg/cm$^2$ for 30 min with continuous stirring (Baldwin and Moore, 1969*a*). In early tests, homogenization was carried out on cell suspensions in a sucrose buffer, pH 7·4 (Ozer and Wallach, 1967), but in later experiments minced tumour tissue was homogenized in a medium containing $NaHCO_3$ ($1 \times 10^{-3}$ mol/l) and $CaCl_2$ ($2 \times 10^{-3}$ mol/l), pH 7·6. After release of $N_2$ gas, EDTA was added to the homogenate to give a concentration of $1 \times 10^{-3}$ mol/l, and nuclei were removed by centrifugation twice at 600 *g* for 15 min. A total extranuclear membrane (ENM) fraction was isolated from the supernatant by centrifugation at 105,000 *g* for 60 min. The 105,000 *g* supernatant was concentrated by dialysis against Aquacide (Calbiochem Ltd., London, England) to give a soluble "cell sap" fraction.

A plasma membrane fraction was also isolated from tumour cells as described by Baldwin and Moore (1968, 1969*b*). Cells were gently stirred for 16 hours at 2°C in 0·9% w/v NaCl. They were harvested by centrifugation at 600 *g* for 15 min and extracted with distilled water to a point short of cell lysis. Eluted membrane material was collected from the aqueous extract by centrifugation at 80,000 *g* for 30 min.

The protein concentration of subcellular fractions was assayed by the method of Lowry *et al.* (1951).

*Immunogenicity tests.*—Syngeneic rats of the appropriate sex were immunized against extranuclear membrane, plasma membrane and soluble cell fractions by repeated intraperitoneal injections at weekly intervals. Rats were challenged subcutaneously 7–14 days after the final injection with known numbers of tumour cells suspended in medium 199. The growth of tumours in immunized rats was compared with that in untreated controls receiving the same cell inoculum.

In some experiments rats were immunized with heavily irradiated (15,000 rad) tumour cells by 3–8 subcutaneous inoculations at 2-week intervals (Baldwin and Barker, 1967*a*).

*Adoptive transfer tests.*—Immunized and control rats were injected intraperitoneally with 3 ml of a 3% w/v suspension of hydrolysed starch (Connaught Laboratories, Toronto, Canada) in phosphate buffered saline (PBS), pH 7·3. Two days later peritoneal exudate cells were aspirated in medium 199 containing heparin (50 i.u./ml). The cells were washed in 199 and mixed *in vitro* in fixed ratios with known numbers of tumour cells, and the mixtures were immediately injected subcutaneously into syngeneic rats. The peritoneal cells were 100% viable and consisted of about 80% macrophages, 15–18% lymphocytes and a small propor- of granulocytes.

*Immunofluorescence test.*—A membrane immunofluorescence test was employed to detect tumour specific humoral antibody in the sera of immunized rats, as previously described (Baldwin and Barker, 1967*b*; Baldwin *et al.*, 1971). A fluorescence index (FI) was calculated as the proportion of unstained cells in samples exposed to normal rat serum, minus the proportion of unstained cells in the sample exposed to test serum, divided by the former figure. An FI of 0·30 or greater represents a statistically significant positive reaction.

*Colony inhibition tests.*—Colony inhibition (CI) tests were carried out to detect lymph node cell (LNC) mediated immunity and complement dependent cytotoxic antibody against hepatoma cells, as described by Baldwin and Embleton (1971). Target cells were hepatoma cell lines serially propogated *in vitro* as monolayers in Eagle's minimum essential medium (MEM) supplemented with calf serum (10%), penicillin (200 i.u./ml) and streptomycin (100 $\mu$g/ml).

In addition to studying LNC and antisera separately, the blocking effect of sera from ENM immunized rats on colony inhibition mediated by LNC from rats immunized with heavily irradiated tumour grafts was examined. In these tests, target cells (1 to $2 \times 10^3$) were plated in 4 ml of medium into 5 cm plastic petri dishes and incubated for 24 hours at 37°C. The medium was removed and replaced by either normal rat serum or test serum (0·1 ml) diluted with MEM (0·4 ml). Sera were inactivated at 56°C for 30 min before use. The dishes were incubated at 37°C for 30 min and the serum was removed. LNC ($5 \times 10^6$ cells in 0·5 ml MEM) from normal or immune rats were then added, followed by a further 45 min incubation. MEM containing 10% calf serum (3·5 ml) was added to the LNC in each dish and the cultures were incubated for 6 days. The hepatoma cell colonies developing were fixed in methanol, stained with 1% aqueous crystal violet and counted. Three to 4 dishes were used for each combination of LNC and serum.

The percentage CI in the presence of each serum was calculated by comparing the number of colonies formed in dishes treated with immune LNC with those in dishes treated with normal LNC. Percent abrogation of CI was calculated by comparing %CI in the presence of test serum with %CI in the presence of normal rat serum. The results were evaluated statistically by Student's *t* test.

### RESULTS

### *Tumour rejection tests*

Experiments in which the capacity of subcellular fractions of hepatoma D23 to produce immunoprotection in syngeneic rats against a low-dose ($10^3$ cells) challenge with hepatoma D23 are summarized in Table I. In only one of the 5 experiments with the total extranuclear membrane fraction (ENM) of hepatoma D23 was there any protective response to challenge with hepatoma D23 cells but these rats failed to reject a second challenge with $5 \times 10^3$ tumour cells. Likewise, no protection was produced with a plasma membrane fraction prepared by aqueous elution of hepatoma D23 cells, although both this fraction and the ENM preparations retain hepatoma D23 specific antigen, as assayed by their capacity to

TABLE I.—*Induction of Immunity to Transplanted Hepatoma D23 By Hepatoma D23 Cells and Subcellular Fractions*

| Immunizing fraction | Number of injections | Total dose mg protein | Tumour takes* in Treated | Untreated |
|---|---|---|---|---|
| Extranuclear membrane | 1 | 14·1 | 4/4 | 5/5 |
| | 3 | 69·3 | 4/4 | 6/6 |
| | 7 | 70·1 | 1/6 | 6/6 |
| | 9 | 94·7 | 6/6 | 6/6 |
| | 10 | 181·4 | 2/3 | 4/4 |
| Plasma membrane | 3 | 18·2 | 4/4 | 6·6 |
| | 6 | 18·1 | 2/2 | 3/3 |
| | 6 | 16·9 | 5/5 | 4/4 |
| | 8 | 84·3 | 4/4 | 3/4 |
| Cell sap | 3 | 78·6 | 4/4 | 4/4 |
| | 8 | 42·6 | 4/4 | 5/5 |

* Challenge inoculum 1 × $10^3$ hepatoma D23 cells.

TABLE II.—*Effect of Peritoneal Exudate Cells from Donors Immunized with D23 Extranuclear Membrane Fractions on Growth of Tumour D23 in Normal Syngeneic Recipients*

| Immunization of peritoneal exudate cell donor: No. injections | mg protein | Tumour cell dose | Peritoneal cell dose | Peritoneal : tumour cell ratio | Tumour takes |
|---|---|---|---|---|---|
| 5 × ENMF* | 103·8 | $10^4$ | 4 × $10^5$ | 40 : 1 | 5/5 |
| None | — | $10^4$ | 4 × $10^5$ | 40 : 1 | 5/5 |
| 6 × ENMF | 119·8 | $10^4$ | $10^6$ | 100 : 1 | 5/5 |
| None | — | $10^4$ | $10^6$ | 100 : 1 | 5/5 |
| 4 × IR graft† | — | $10^4$ | 5 × $10^5$ | 50 : 1 | 0/4 |
| 6 × ENMF | 119·8 | $10^4$ | 5 × $10^5$ | 50 : 1 | 4/4 |
| None | — | $10^4$ | 5 × $10^5$ | 50 : 1 | 4/4 |
| 5 × IR graft | — | $10^4$ | 5 × $10^5$ | 50 : 1 | 1/4 |
| 6 × ENMF | 119·8 | $10^4$ | 5 × $10^5$ | 50 : 1 | 4/4 |
| None | — | $10^4$ | 5 × $10^5$ | 50 : 1 | 4/4 |

* ENMF: rats immunized with extranuclear membrane fractions.
† IR graft: rats immunized with irradiated grafts of whole tumour tissue.

absorb antibody from syngeneic tumour immune sera (Baldwin and Moore, 1969*a*). The soluble cytoplasmic fraction isolated from tumour homogenates prepared by nitrogen pressure homogenization also did not elicit tumour immunity and this is consistent with previous studies showing these fractions to be deficient in tumour specific antigen, assayed by their capacity to neutralize tumour specific antibody in syngeneic antisera, as measured by membrane immunofluorescence tests (Baldwin and Moore, 1969*b*). These findings with subcellular fractions contrast with the consistent immunogenicity of intact hepatoma cells where immunization with irradiated cells 1–3 times at 2-weekly intervals provides protection to subsequent challenges with up to 5 × $10^5$ viable hepatoma cells (Baldwin and Barker, 1967*a*).

*Cellular and humoral immune responses to hepatoma D23 subcellular fractions in syngeneic rats*

Cell mediated immunity elicited against hepatoma D23 ENM was assayed by the capacity of peritoneal exudate (PE) cells from treated rats to adoptively transfer resistance (Table II). In agreement with

TABLE III.—*Inhibition of Hepatoma Cell Colony Formation by Lymph Node Cells or Serum from ENM Immunized Rats*

| Target cells | ENM treatment of LNC and serum donors: No. injections | Mg protein | % Colony inhibition†, ‡ in the presence of: Lymph node cells | | | Serum | | |
|---|---|---|---|---|---|---|---|---|
| D23 | 5 | 103·8 | 90·5*** | 70·1*** | | 97·0*** | 98·5*** | |
| | | | 79·6*** | 8·6 | | −5·8 | | |
| D23 | 6 | 119·8 | −15·3 | 45·0 | | 39·4*** | 40·1*** | 35·6** |
| | | | −18·6 | | | 42·0*** | 74·5*** | |
| D23 | 8 | 204·0 | −3·9 | 11·5 | −7·6 | 74·5*** | | |
| | | | −3·9 | −5·6 | | | | |
| D23 | 4 | 78·2 | | | | 72·3** | 63·8** | |
| D23 | 5 (D30)§ | 85·3 | 6·1 | | | | | |
| D30 | 4 | 39·8 | −3·8 | 5·1 | −3·8 | 5·2 | | |
| | | | −10·8 | 15·9* | | | | |
| D30 | 5 | 19·6 | 5·9 | 5·9 | 10·6* | 4·7 | | |
| D30 | 5 | 85·3 | | | | 1·3 | | |
| D30 | 6 | 43·3 | 19·0* | 15·4** | | 6·5 | 2·4 | |
| | | | | | | 2·0 | 15·0** | |
| D31 | 4 | 46·2 | | | | −2·6 | 4·0 | 1·7 |
| D31 | 5 | 47·6 | −10·0 | −3·6 | 14·6 | 14·3* | 5·3 | −10·8 |
| D31 | 6 | 57·6 | 1·8 | 18·2* | | | | |
| D31 | 6 (D23)§ | 119·8 | −19·2 | | | | | |

§ % colony inhibition = mean % reduction of colony numbers in dishes treated with test LNC or serum, compared with those treated with control LNC or serum.

† Probability that % colony inhibition values are due to chance is indicated by: $^{*}P < 0 \cdot 05$ $^{**}P < 0 \cdot 01$ $^{***}P < 0 \cdot 001$.

‡ Rats treated with ENM from a tumour different from the target cells.

a previous study (Baldwin and Barker, 1967*b*) peritoneal exudate cells from rats immunized against hepatoma D23 suppressed tumour growth in all but one rat when injected in ratios of 50/tumour cell. Peritoneal exudate cells from ENM treated rats at doses up to 100 PE cells/tumour cell were completely ineffective, so that tumours developed in all treated rats and at rates comparable with those in rats receiving PE cells from normal animals.

Cellular immunity elicited by hepatoma D23 ENM preparations was also examined *in vitro* by the capacity of lymph node cells (LNC) from treated rats in comparison with LNC from untreated controls to inhibit colony formation of plated hepatoma D23 cells (Table III). Of the 13 preparations tested, only 3 produced significant inhibition of colony formation so that the LNC mediated response to hepatoma D23 ENM was much less consistent than that elicited by intact cells (Baldwin and Embleton, 1971). It is notable, however, that in the 3 positive tests the LNC exerted a pronounced inhibitory effect, producing colony inhibition indices of 70·1–90·5%. ENM preparations from 2 other hepatomata, D30 and D31, were also examined for their capacity to elicit the formation of sensitized LNC which inhibit colony formation by cells of the homologous tumour (Table III). Only 4/10 of the tests with LNC from hepatoma D30 ENM treated rats and 1/6 tests with hepatoma D31 ENM treated rats produced significant inhibition of colony formation. Moreover, these reactions were much weaker in terms of the percentage colony inhibition compared with the reactivity of LNC from rats immunized against intact hepatoma cells (Baldwin and Embleton, 1971).

### *Humoral antibody*

Tumour specific antibody elicited against hepatoma D23 subcellular fractions was assayed by the capacity of sera from treated rats to react in immunofluorescence tests with cell membrane expressed antigen on viable hepatoma

TABLE IV.—*Humoral Antibody Response Evoked by Cells and Subcellular Fractions of Hepatoma D23*

| Expt No. | Hepatoma* fraction | No. of injections | Total immunizing dose (mg protein) | Fluorescence index† against D23 cells |
|---|---|---|---|---|
| 1A | Whole cells | 3 | Graft | 0·37, 0·48, 0·53, 0·45 |
| 2B | Whole cells | 8 | Graft | 0·68, 0·50 |
| 2A | Extranuclear membrane | 1 | 14·1 | 0·02, 0, 0·08, 0·01, 0·32, 0·07 |
| 2B | Extranuclear membrane | 3 | 40·0 | 0·43, 0·24, 0·66, 0·37, 0·48 |
| 2C | Extranuclear membrane | 7 | 70·1 | 0·53, 0·32, 0·37, 0·72, 0·30 |
| | Extranuclear membrane | 3 | 40·0 | 0·30, 0·26, 0·63, 0·63, 0·52 |
| 2D | Extranuclear membrane | 5 | 110·5 | 0·48, 0·39, 0·68, 0·59, 0·59 |
| 2E | Extranuclear membrane | 3 | 69·3 | |
| 3A | Plasma membrane | 4 | 56·9 | 0·53, 0·55 |
| | Plasma membrane | 8 | 84·3 | 0·70, 0·38, 0·75, 0·81 |
| 3B | Plasma membrane | 6 | 16·9 | 0·81, 0·79, 0·72, 0·65, 0·73 |
| 3C | Plasma membrane | 6 | 18·1 | 0·46, 0·72, 0·46, 0·36 |
| 3D | Plasma membrane | 3 | 18·2 | |
| 4A | Soluble | 3 | 78·6 | 0·14, 0·04, 0, 0, 0·10, 0·16 |
| 4B | Soluble | 8 | 42·6 | 0·04, 0·15 |

* Whole cells received 15,000 rad $^{60}$Co $\gamma$-irradiation.
† A fluorescence index of 0·30 or greater represents a positive reaction.

D23 cells in suspension (Table IV). Both types of hepatoma D23 membrane preparation (ENM and plasma membrane) elicited good tumour specific antibody responses, fluorescence indices of between 0·30 and 0·81 being obtained with antisera. Predictably, a single immunization with ENM did not elicit detectable levels of tumour specific antibody but it is significant that where multiple doses of either preparation were administered, even small amounts (16·9 mg protein) of membrane produced sera with fluorescence indices even higher than those observed with sera from rats immunized against irradiated tumour cells or by excision of tumour grafts and multiple challenges with viable tumour cells (Baldwin and Barker, 1967*b*; Baldwin *et al.*, 1971). These qualitative data therefore indicate that the hepatoma D23 membrane preparations are as good as, or even more effective than, intact cells in eliciting tumour specific antibody responses in syngeneic rats. In contrast, the cytoplasmic fraction which is considered to be lacking in tumour specific antigen (Baldwin and Moore, 1969*a*) did not induce antibody responses detectable by membrane immunofluorescence staining of viable hepatoma D23 cells (Table IV).

Antibody elicited against hepatoma D23 ENM was also demonstrated by the complement dependent cytotoxicity of sera from treated rats for plated hepatoma D23 cells (Table III). In this case the expression of cytotoxic antibody (10/11 tests positive, CI 35·6–98·5%) was a much more frequent event than the development of cytotoxic sensitized LNC. The formation of tumour specific antibody appeared to be a specific feature of hepatoma D23 ENM since in tests with ENM preparations from hepatoma D30 and D31 only 2/13 sera were cytotoxic for cells of the homologous tumour (CI 15·0 and 14·3% respectively).

*Blocking of lymph node cell cytotoxicity for hepatoma cells by rat antisera against tumour ENM fractions*

Tests were carried out to evaluate whether sera prepared by treating rats with ENM fractions from hepatomata

TABLE V.—*Abrogation of Cell Mediated Inhibition of Hepatoma Cell Colony Formation by Sera from ENM Immunized Rats*

| Target cells | Immunization of LNC donor* | ENM treatment of serum donor: No. injections | ENM treatment of serum donor: Mg protein | % Colony† Inhibition | $P <$ | %‡ Abrogation | $P <$ |
|---|---|---|---|---|---|---|---|
| D23 | 3 IR + 3 ch | None | None | 66·7 | 0·001 | | |
| D23 | 3 IR + 3 ch | 6 | 119·8 | 6·1 | 0·30 | 90·7 | 0·001 |
| D23 | 3 IR + 3 ch | 6 | 119·8 | 2·1 | 0·35 | 96·7 | 0·001 |
| D23 | 3 IR + 3 ch | 5 (D30) | 85·3 | 72·4 | 0·005 | −9·2 | |
| D23 | 4 IR + 2 ch | None | None | 35·9 | 0·005 | | |
| D23 | 4 IR + 2 ch | 5 | 103·8 | 17·2 | 0·01 | 52·1 | 0·05 |
| D23 | 4 IR + 8 ch | None | None | 18·6 | 0·05 | | |
| D23 | 4 IR + 8 ch | 8 | 204 | 0 | | 100 | 0·05 |
| D23 | 3 IR + 6 ch | None | None | 21·5 | 0·005 | | |
| D23 | 3 IR + 6 ch | 8 | 204 | 9·9 | 0·05 | 53·9 | 0·20 |
| D30 | 3 IR + 3 ch | None | None | 20·3 | 0·05 | | |
| D30 | 3 IR + 3 ch | 4 | 39·8 | 13·2 | 0·25 | 34·7 | 0·20 |
| D30 | 3 IR + 3 ch | 5 | 29·6 | 10·4 | 0·20 | 48·7 | 0·20 |
| D31 | 3 IR + 8 ch | None | None | 46·1 | 0·05 | | |
| D31 | 3 IR + 8 ch | 4 | 46·2 | −21·3 | | 100·0 | 0·05 |
| D31 | 3 IR + 8 ch | 5 | 47·6 | −13·9 | | 100·0 | 0·05 |
| D31 | 4 IR + 2 ch | None | None | 16·9 | 0·05 | | |
| D31 | 4 IR + 2 ch | 4 | 46·2 | −7·4 | | 100·0 | 0·01 |
| D30 | 14 days post-exc. | None | None | 29·7 | 0·05 | | |
| D30 | 14 days post-exc. | 6 | 35·0 | −12·6 | | 100·0 | 0·05 |
| D30 | 14 days post-exc. | 4 (D31) | 46·2 | 20·7 | 0·05 | 30·4 | 0·45 |
| D31 | 21 days post-exc. | None | None | 26·9 | 0·01 | | |
| D31 | 21 days post-exc. | 4 | 46·2 | 0 | | 100·0 | 0·001 |
| D31 | 21 days post-exc. | 5 | 46·2 | 2·0 | 0·475 | 92·6 | 0·01 |
| D31 | 21 days post-exc. | 4 (D30) | 39·8 | 37·2 | 0·01 | −38·4 | |

* IR = treatment with irradiated (15,000 rad $^{60}Co$ $\gamma$-irradiation) tumour grafts; ch = challenge with viable hepatoma cells; post-exc. = removal of growing tumour by surgical excision.

† % colony inhibition = % reduction of colony numbers in dishes treated with immune LNC, compared with those treated with control LNC.

‡ % abrogation = % reduction of LNC mediated colony inhibition in dishes treated with test serum compared with % colony inhibition in those treated with control serum.

D23, D30 and D31 could specifically protect the homologous target tumour cells from the cytotoxic action of sensitized lymph node cells (Table V). The sensitized LNC used in each test were from rats immunized by repeated implantation of irradiated tumour followed by a series (2–8) of challenges with viable tumour cells. In the case of hepatomata D30 and D31 LNC were also taken from rats 14 and 21 days respectively after excision of hepatoma grafts. In the tests with hepatoma D23, pretreatment of plated tumour cells with normal serum did not abolish their susceptibility to hepatoma D23 sensitized lymph node cells and in each of the 4 tests significant inhibition of colony formation was obtained (CI 18·6–66·7%). In the first experiment pretreatment of plated hepatoma D23 cells with D23 ENM immune sera reduced the CI from 66·7% to 6·1 and 2·1% respectively, this representing 90·7–96·7% abrogation of LNC cytotoxicity. In contrast, treatment with hepatoma D30 ENM antiserum had no effect so that the colony inhibition was slightly greater (72·4%) than that observed with cells treated with normal serum. This is consistent with the known immunological specificities of the tumour antigens associated with these hepatomata and indicates that the blocking action of ENM antisera is a specific event.

Pretreatment of plated tumour cells with sera from rats immunized against ENM preparations of hepatoma D30 and D31 also specifically inhibited the cytotoxicity of sensitized LNC. In tests with hepatoma D30 pretreatment of plated hepatoma cells with 3 different sera from rats immunized with ENM fractions of the

TABLE VI.—*Impairment of Host Resistance to Hepatoma D23 by Pretreatment of Rats with Hepatoma D23 Membrane*

| D23 membrane pretreatment | | | | | | Challenge takes in: | |
|---|---|---|---|---|---|---|---|
| No. of injections | Mg protein | Immunizing tissue | No. of IR* grafts | Challenge tumour | No. of cells | Treated rats | Controls |
| 6 | 52·3 | Normal liver | 2 | D23 | $10^3$ | 5/5 | 6/6 |
| 6 | 52·3 | D23 | 2 | D23 | $10^3$ | 4/5 | 6/6 |
| None | — | D23 | 2 | D23 | $10^3$ | 0/5 | 6/6 |
| 5 | 69·7 | Normal liver | 2 | D33 | $2 \times 10^4$ | 4/4 | 4/4 |
| 5 | 69·7 | D33 | 2 | D33 | $2 \times 10^4$ | 0/5 | 5/5 |
| None | — | D33 | 2 | D33 | $2 \times 10^4$ | 1/5 | 5/5 |

* IR: Irradiated tumour graft (15,000 rad) $^{60}$Co $\gamma$-irradiation.

homologous tumour reduced LNC mediated colony inhibition to an insignificant level. These tests included a cross test in which plated hepatoma D30 cells were treated with serum from rats immunized with D31 ENM but this did not produce significant blocking. All of the sera prepared against hepatoma D31 fractions were highly effective in blocking the cytotoxicity of sensitized LNC for plated hepatoma D31 cells. In one experiment 2 sera against hepatoma D31 ENM produced 100% and 92·6% blocking of D31 sensitized LNC cytotoxicity, whereas sera from D30 ENM immunized rats were without effect.

*Impairment of host resistance to hepatoma D23 following pretreatment of rats with hepatoma D23 membrane*

These experiments were designed to ascertain whether immunization against hepatoma D23 ENM, which preferentially elicits a humoral immune response, would modify the tumour rejection response elicited by irradiated hepatoma cells. (Table VI). Rats pretreated with hepatoma D23 ENM, and subsequently immunized with irradiated hepatoma D23 grafts, were not able to reject a challenge with hepatoma D23 cells which developed into progressively growing tumours in all recipients. Likewise, immunization with D23 ENM followed by irradiated normal liver failed to induce any immunity against hepatoma D23. Control rats receiving only irradiated hepatoma D23 cells all elicited a specific resistance against hepatoma D23 cells. The second experiment was carried out as a specificity control and in this case an initial immunization with hepatoma D23 ENM did not influence subsequent immunization with irradiated grafts of hepatoma D33.

### DISCUSSION

The principal expression of immunity elicited in syngeneic rats by hepatoma membrane fraction was the development of a humoral antibody response, demonstrable by membrane immunofluorescence staining of viable hepatoma cells, and by complement dependent cytotoxicity of the sera for plated hepatoma cells, resulting in a significant inhibition of colony formation. The cell mediated immune response, on the other hand, was generally weak so that lymph node cells from membrane immunized rats did not consistently produce a significant inhibition of colony formation by plated hepatoma cells. Also, peritoneal exudate cells from these rats were uniformly ineffective in adoptively transferring immunity to normal rats.

Correlated with the overall immune response elicited by hepatoma D23 membrane immunization, in which cellular immunity was not a prominent feature, was a failure of treated rats to reject challenge with threshold doses ($1 \times 10^3$) of viable hepatoma cells. This contrasts with reproducible induction of tumour resistance following excision of hepatoma grafts; rats treated in this manner rejecting up to 500-fold larger challenge with hepatoma cells (Baldwin and Barker, 1967*a*). With this form of immunization there is an effective cell mediated immune response so that lymph node cells taken from rats post-excision inhibit colony formation, or cell survival as assayed by the microcytotoxicity test (Baldwin *et al.*, 1973*b*). Cytotoxic antibody is also demonstrable in post-excision sera, but these sera do not show positive immunofluorescence staining with viable hepatoma cells. Rats immunized by repeated implantation of $^{60}$Co $\gamma$-irradiated hepatoma also specifically reject subsequent challenges with cells of the same tumour. This form of immunization again provokes a significant cellular immune reaction and in this case, tumour-specific antibody is demonstrable both by serum cytotoxicity and membrane immunofluorescence reactions (Baldwin and Barker, 1967*b*; Baldwin and Embleton, 1971; Baldwin *et al.*, 1973).

The failure of hepatoma membrane immunization to evoke tumour rejection reactions, therefore, appears to be associated with an immune deviation favouring the formation of tumour specific antibody. This is further emphasized by the finding that serum from rats immunized with hepatoma membrane specifically blocks hepatoma cells *in vitro* from cytotoxic attack by lymph node cells from tumour immune donors. Even more important than this effect at the efferent level was the observation that prior immunization with hepatoma D23 membrane specifically impairs the capacity of rats to elicit a tumour rejection response on subsequent immunization with irradiated tumour, pointing to a regulatory effect on the lymphoid system.

These observations are pertinent in respect of current developments, suggesting that interference by circulating humoral factors, including tumour antigen, antibody and immune complexes, on cell mediated immunity is of paramount importance in the outcome of a tumour immune response (Baldwin *et al.*, 1972, 1973*b*). Sera from rats bearing transplants of hepatoma D23, for example, specifically block hepatoma D23 cells *in vitro* from attack by sensitized lymph node cells. These sera will also directly inhibit the reactivity of lymph node cells so that preincubation of serum with effector cells for a short period, and subsequent washing, reduce their cytotoxicity *in vitro* for hepatoma D23 cells (Baldwin *et al.*, 1973*a*). Blocking at the level of the target cells, it is postulated, is mediated by tumour specific immune complexes since these sera have been shown to contain such factors in a region of antigen excess (Baldwin *et al.*, 1973). Moreover, the blocking effect of immune complexes has been established in model studies using complexes prepared by addition of papain solubilized hepatoma D23 antigen and post-excision sera as a source of tumour specific antibody (Baldwin *et al.*, 1972). Direct inhibition of lymphocyte reactivity can be effected by solubilized hepatoma D23 antigen (Baldwin *et al.*, 1973*b*) so that the effect observed with tumour bearer serum may also be induced by circulating antigen or immune complexes. When these humoral factors are analysed in relation to tumour status, it is found that following surgical removal of tumour the serum factors producing tumour cell blocking and lymphocyte inhibition are rapidly lost, and at this stage tumour specific cytotoxicity antibody becomes demonstrable (Baldwin *et al.*, 1973). On subsequent repeated immunization, tumour immune sera continue to demonstrate complement dependent cytotoxicity and antibody is demonstrable by membrane immunofluorescence. At this stage these antisera, in the absence of complement show blocking activity, protecting hepatoma cells *in vitro* from sensitized lymph node cells, but do not directly inhibit lymph node cell reactivity (Baldwin *et al.*, 1973*a*, *b*). Within this context, antibody elicited by hepatoma D23 membrane may act directly as " blocking antibody ", protecting hepatoma D23 cells from lymphocyte attack. Even though antibody is present in excess, immune complexes may also be present, resulting from binding with solubilized hepatoma D23 antigen released from the immunizing inoculum. This may explain why antibody was not regularly demonstrable in sera from rats immunized with hepatomata D30 and D31 membrane although the sera consistently blocked tumour cells from attack by sensitized lymph node cells. The release of antigen into the circulation from the immunizing membrane may also produce an inhibition of the lymphocyte response. This may be effected by tumour antigen inhibition of stimulated lymphocytes, as demonstrated in model studies showing that the cytotoxicity of lymph node cells from hepatoma D23 immune rats can be totally inhibited by addition of papain solubilized antigen (Baldwin *et al.*, 1973*b*). Additionally, circulating tumour antigen may act to inhibit the production of sensitized lymphocytes in a manner analogous to the observed lymphocyte anergy in local nodes draining a tumour (Alexander *et al.*, 1969).

These studies with transplanted rat hepatomata indicate the limitations of using antigenically active subcellular tumour fractions for tumour immunization. Comparably, it has been established that immunization with membrane fractions from 3-methylcholanthrene induced sarcomata and a spontaneous mammary carcinoma (Baldwin and Embleton, 1970; Baldwin, Pimm and Price, to be published) does not result in the development of a tumour rejection reaction. It cannot be generalized from these studies, however, that tumour fractions cannot be used to induce tumour immunity since, for example, injection of soluble extracts of guinea-pig sarcomata induced a degree of resistance comparable with that obtained with intact cells (Holmes *et al.*, 1970; Suter *et al.*, 1972). The conditions governing the type of response elicited against different tumours in a variety of species have yet to be defined but one important condition is likely to be the degree of stability of the tumour antigen within the cell membrane. The present observations are also pertinent to studies now in progress attempting to utilize immunostimulants such as B.C.G. for immunotherapy. For example, with Moloney sarcomata in the rat, B.C.G. immunostimulation before, or at the time of, tumour transplantation produces an immunoprotective response, whereas when given 2 weeks after, tumour enhancement is obtained (Bansal and Sjögren, 1973). Under the latter condition it is highly probable that in addition to the local tumour mass soluble or particulate tumour antigen will be present in the circulation and from the rat hepatoma studies reported here, the type of immune response will be different from that arising where only local tumour is present, favouring a humoral antibody response. Although this has not been analysed in depth, one consequence of B.C.G. in rats bearing established Moloney sarcomata is an increase in serum blocking factors, which is consistent with the findings from the present studies with rat hepatomata showing enhanced antibody levels.

This work was supported by the Cancer Research Campaign.

### REFERENCES

ALEXANDER, P., BENSTED, J., DELORME, E. J., HALL, J. G. & HODGETT, J. (1969) The Cellular Immune Response to Primary Sarcomata in Rats. II. Abnormal Responses of Nodes draining the Tumour. *Proc. R. Soc. B.*, **174**, 237.

BALDWIN, R. W. & BARKER, C. R. (1967*a*) Tumour-specific Antigenicity of Aminoazo Dye-Induced Rat Hepatomas. *Int. J. Cancer*, **2**, 355.

BALDWIN, R. W. & BARKER, C. R. (1967*b*) Demonstration of Tumour-specific Humoral Antibody against Aminoazo-Dye-Induced Rat Hepatomata. *Br. J. Cancer*, **21**, 793.

BALDWIN, R. W., BARKER, C. R., EMBLETON, M. J., GLAVES, D., MOORE, M. & PIMM, M. V. (1971) Demonstration of Cell-surface Antigens on Chemically Induced Tumors. *Ann. N.Y. Acad. Sci.*, **177**, 268.

BALDWIN, R. W., BOWEN, J. G. & PRICE, M. R. (1973) Detection of Circulating Hepatoma D23 Antigen and Immune Complexes in Tumour Bearer Serum. *Br. J. Cancer*, **28**, 16.

BALDWIN, R. W. & EMBLETON, M. J. (1970) Detection and Isolation of Tumour-specific Antigen Associated with a Spontaneously Arising Rat Mammary Carcinoma. *Int. J. Cancer*, **6**, 373.

BALDWIN, R. W. & EMBLETON, M. J. (1971) Demonstration by Colony Inhibition Methods of Cellular and Humoral Reactions to Tumour Specific Antigens Associated with Aminoazo Dye-Induced Rat Hepatomas. *Int. J. Cancer*, **7**, 17.

BALDWIN, R. W., EMBLETON, M. J. & ROBINS, R. A. (1973) Cellular and Humoral Immunity to Rat Hepatoma-specific Antigens Correlated with Tumour Status. *Int. J. Cancer*, **11**, 1.

BALDWIN, R. W. & GLAVES, D. (1972) Solubilization of Tumour-specific Antigen from Plasma Membrane of an Aminoazo Dye-Induced Rat Hepatoma. *Clin. & exp. Immunol.*. **11**, 51.

BALDWIN, R. W., HARRIS, J. R. & PRICE, M. R. (1973) Fractionation of Plasma Membrane-associated Tumour-specific Antigen from an Aminoazo Dye-induced Rat Hepatoma. *Int. J. Cancer*, **11**, 385.

BALDWIN, R. W. & MOORE, M. (1968) Isolation of Membrane-associated Tumour-specific Antigens from Rat Hepatomas Induced by Aminoazo Dye. *Nature, Lond.*, **220**, 287.

BALDWIN, R. W. & MOORE, M. (1969*a*) Isolation of Membrane Associated Tumour-specific Antigen from an Aminoazo-dye-induced Rat Hepatoma. *Int. J. Cancer*, **4**, 753.

BALDWIN, R. W. & MOORE, M. (1969*b*) Rat Hepatoma Cell Surface Antigens: Demonstration and Isolation of Membrane-associated Isoantigens. *Eur. J. Cancer*, **5**, 475.

BALDWIN, R. W., PRICE, M. R. & ROBINS, R. A. (1972) Blocking of Lymphocyte-mediated Cytotoxicity for Rat Hepatoma Cells by Tumour-specific Antigen–Antibody Complexes. *Nature, New Biol.*, **238**, 185.

BALDWIN, R. W., PRICE, M. R. & ROBINS, R. A. (1973*a*) Inhibition of Hepatoma Immune Lymph Node Cell Cytotoxicity by Tumour Bearer Serum, and Solubilized Hepatoma Antigen. *Int. J. Cancer*, **11**, 527.

BALDWIN, R. W., PRICE, M. R. & Robins, R. A. (1973*b*) Significance of Serum Factors Modifying Cellular Immune Responses to Growing Tumours. In: M. Moore, N. W. Nisbet & M. V. Haigh (eds.) *Immunology of Malignancy. Br. J. Cancer*, **28** *Suppl.* 1, 37.

BANSAL, S. C. & SJÖGREN, H. O. (1973) Effects of BCG on Various Facets of the Immune Response against Polyoma Tumours in Rats. *Int. J. Cancer*, **11**, 162.

HOLMES, E. C., KAHAN, B. D. & MORTON, D. L. (1970) Soluble Tumor-specific Transplantation Antigens from Methylcholanthrene-induced Guinea Pig Sarcomas. *Cancer, N.Y.*, **25**, 373.

LOWRY, O. H., ROSEBROUGH, N. J., FARR, A. L. & RANDALL, R. J. (1951) Protein Measurements with the Folin Phenol Reagent. *J. biol. Chem.*, **193**, 265.

MELTZER, M. S., LEONARD, E. J., RAPP, H. J. & BORSOS, T. (1971) Tumor-specific Antigen Solubilized by Hypertonic Potassium Chloride. *J. natn. Cancer Inst.*, **47**, 703.

OETTGEN, H. F., OLD, L. J., MCLEAN, E. P. & CARSWELL, E. A. (1968) Delayed Hypersensitivity and Transplantation Immunity Elicited by Soluble Antigens of Chemically-induced Tumors in Inbred Guinea Pigs. *Nature, Lond.*, **220**, 295.

OZER, J. H. & WALLACH, D. F. H. (1967) H-2 Components and Cellular Membranes: Distinctions between Plasma Membrane and Endoplasmic Reticulum Governed by the H-2 Region in the Mouse. *Transplantation*, **5**, 652.

SUTER, L., BLOOM, B. R., WADSWORTH, E. M. & OETTGEN, H. F. (1972) Use of the Macrophage Migration Inhibition Test to Monitor Fractionation of Soluble Antigens of Chemically Induced Sarcomas of Inbred Guinea Pigs. *J. Immun.*, **109**, 766.

THOMSON, D. M. P. & ALEXANDER, P. (1973) A Cross-reacting Embryonic Antigen in the Membrane of Rat Sarcoma Cells which is Immunogenic in the Syngeneic Host. *Br. J. Cancer*, **27**, 35.

THOMSON, D. M. P., STEEL, K. & ALEXANDER, P. (1973) The Presence of Tumour-specific Membrane Antigen in the Serum of Rats with Chemically Induced Sarcomata. *Br. J. Cancer*, **27**, 27.